# Role of ‘One Stop Crisis Centre’ in Identifying and Assisting Victims of Violence in an Indian Health Care Setup

**DOI:** 10.5195/cajgh.2020.297

**Published:** 2020-03-31

**Authors:** Prachi Verma, Payal Puri, Dhruv Sharma, Shreya Singh

**Affiliations:** 1 School of Management Studies, Punjabi University, Patiala, India; 2 District Hospital, Panchkula, India

**Keywords:** Violence against women, Victims of violence, One Stop Crisis Centre, Screening, North India

## Abstract

**Introduction:**

Cases of female targeted violence often go uncounted in India. To identify the unreported cases of violence, Sukoon was established in 2014 as a hospital-based ‘One Stop Crisis Centre’ (OSCC). Sukoon provides counselling, police assistance and legal aid to the victims. The aim of the present study was to recognize the role of Sukoon in preventing violence against women (VAW) in the region.

**Methods:**

Secondary data was extracted from 430 victims who approached Sukoon from August 2014 through January 2017. Data was collected on different variables: age, marital status, nature of violence, medium through which victims approached Sukoon and type of assistance provided. Significance of association of studied factors with the type of assault was investigated using χ^2^ test.

**Results:**

Age of study-victims ranged from 4 to 75 years with a median age of 26 years and mean age of 27.61 years with standard deviation of 10.56 years. Major types of VAW (96.51%) were domestic violence, sexual assault, physical assault and poisoning. The types of violences were significantly associated with victims’ age (χ^2^ =5.76, d.f.=1, p<0.05) and marital status (χ^2^ = 98.23, d.f=4, p<0.001). About 78% of victims were identified from Sukoon through screening and counseling. Around 69% of the cases were resolved directly by Sukoon or through police assistance.

**Conclusions:**

The above results indicate a significant role of Sukoon in screening victims of violence and providing them required assistance within the hospital environment in one location. Such centers should be further promoted by the government to address the issues of VAW.

The Indian culture is male dominated, and violence is used as a power to control and discipline women^1^. It is estimated that almost 35% of females experience abuse to physical, social and psychological violence at some point of life^2^. Domestic violence is especially prevalent in Indian society and usually, most of the violence is inflicted by husbands to control their wives^3^. Major risk factors include, alcohol consumption by husbands, poor socioeconomic status, lower level of education, harassment for dowry^4^, family history of violence, age, marriage, size & type of family, culture and caste^5^^–^^7^. Domestic violence in India is believed to be an individual's personal issue^8^. Thus, it is usually accepted and allowed to become a norm of married life or a husband's right^9^. Various social stigmas and psychological indoctrination of the society prevent women from seeking any help, according to studies conducted in other countries^9^^–^^14^. About 75–86% of the women in India do not report that they are victims of violence^15^. According to the report from National Crime Records Bureau (NCRB)^16^, crime against women in India rose to 55.2% in 2016. The crimes included cases of cruelty by husband or his relatives (32.6%), followed by sexual assault (25.0%), kidnapping (19.0%) and rape (11.5%)^16^. Violence against women (VAW) is a growing problem in India but varies across the regions. The state of Haryana has the sixth highest rate of crime against women as per the latest NCRB 2016 data^16^. According to National Family Health Survey Report, about 34% of the women in Haryana aged 15–49 years^17^ have experienced physical or sexual violence. Of this, only 14% have ever sought any help regarding the abuse^17^.

India's first hospital-based crisis center was established in 2001 by the Center for Enquiry into Health and Allied Themes (CEHAT) at the KB Bhabha Hospital, Bandra. It is named Dilaasa, which means ‘reassurance’, and is broadly accepted as the Dilaasa Model^18^,^19^.

The success of the Dilaasa Model laid the foundation for developing ‘One Stop Crisis Centres’ in India by the Ministry of Women and Child Development20 in 2013–2014. In Haryana, the hospital-based ‘One Stop Crisis Centre’ was named Sukoon. Its aim was to offer shelter along with police, legal, medical and counseling services to victims of violence under one roof—incorporated with a 24-hours operational, public, police and Sukoon helpline^20^. After screening victims of violence, further help is given only after the victim provides consent. If the victim complains of sexual assault or attempted rape, screening of the victims is done according to the 'safe kit’ developed by CEHAT^21^. The formally trained counselors by CEHAT play a key role in coordinating and managing all the activities of Sukoon. They instill confidence and support victims, provide assistance in seeking justice and coordinate between victim, hospital, police and legal cell at each step. Families of the victims are also called to Sukoon, and discussions are held by the staff to identify the reasons leading to violence. Trained counselors hold sessions with victims and their families for various time periods. If families give some positive response, and no further violence is reported by the victim in follow up sessions up to one year later and no legal help of any kind was sought by the case, then the case is deemed as resolved. In cases where families were not cooperative, police have to be contacted by the Sukoon staff to intervene in the matter. As per the policy of the Sukoon, for cases which are considered to be resolved, victims are still contacted by the counselors at frequent intervals to check for further acts of violence up to one year. After this, if required, victims can approach Sukoon anytime for further assistance.

To study the role of Sukoon in identifying and assisting the victims of violence, this study was conducted with three objectives. First, to identify the various categories of assaults experienced by the victims. Second, to find out the various ways through which victims were approaching Sukoon, and third, to identify type and status of help received by the victims through Sukoon.

## Methods

Sukoon maintains regular computerized records of victims. All registered victims approached Sukoon through helpline numbers, were identified from the local district hospital, or brought to Sukoon by police. We obtained the records of the center for a period of 2.5 years from August 1, 2014 to January 31, 2017 for this study. During the above period, 430 victims were registered with the center to use the OSCC services. While collecting data from the records of Sukoon, adequate confidentiality was maintained and accordingly, identities of the victims were not disclosed to the public. This study was approved by the Hospital Ethics Committee. For study purposes, the categorization of victims has been explained under various headings, as given below.

Domestic Violence: It is the physical abuse of females within a domestic setting. It may involve physical beating or verbal, emotional, economic and religious abuse.

Sexual Assault: This is an act in which a female is physically abused against her will in any environment. It may include sexual touching, kissing, fondling or attempted rape.

Physical Assault: Any physical attack on a female outside the domestic setting is termed as physical assault. It may be done by an individual or a group of people. It may include pushing, stalking, threatening or harming with a weapon.

Poisoning: A condition where the victim has been given poison as a result of any type of assault with the intention to kill her.

Burn: Where an attempt has been made to burn the victim using any flammable substance by the abusing person.

Attempted Suicide: A situation where a victim tries to end her life by consuming poison or any other means but has survived.

Others: This category includes any other type of violence which is not included above but present in the society, e.g. acid attack, trafficking, etc.

### Statistical Analysis

Victims were categorized in 5 major categories: type of violence experienced, age, marital status, source of entry into the Sukoon Centre and agency involved in resolving the violence-case. The collected data was then analyzed using statistical software SPSS 20. As the data is categorical, Pearson's Chi square test was used to look at the relationship between two pairs of variables. The analysis focused on identifying major types of assaults, studying the role of variables, and investigating the agencies involved in resolving a violence case. Significance of association of studied factors with type of assault was investigated using χ^2^ test due to large sample size. Yates has suggested a correction for continuity in χ^2^ value in case of 2×2 tables, preferably when cell frequencies are smaller than 5; this is popularly known as the Yates correction. Thus, to apply the χ^2^ test at places where cell frequencies were less than 5, either the required number of rows and columns were clubbed together or the well-known Yates correction for 2×2 contingency tables was employed to the test. Because 18 is the cut off point for categorizing an individual as a minor or major, victims were divided into 2 groups: below 18 years (as a minor) and above 18 years for studying the association of age with the types of violence.

## Results

Victims (N=430) were first categorized into different groups, based on the type of violence. On further analysis (Table 1), it was found that a large number of victims, had experienced domestic violence (46.51%), followed by sexual assault (21.86%), poisoning (16.28%), burn (1.63%), other miscellaneous type of violence (1.16%) and suicide (0.70%).

**Table 1.** Types of violence against women (VAW) amongst women approaching Sukoon Centre

Violence type	Violence cases studied	Percentage	Cumulative Percentage
Domestic violence	200	46.51	46.51
Sexual violence	94	21.86	68.37
Physical assault	51	11.86	80.23
Poisoning	70	16.28	96.51
Suicide	3	0.70	97.21
Burn	7	1.63	98.84
Miscellaneous	5	1.16	100.00
Total	430	100.0	

Age and marital status were hypothesized to be associated with the type of assault, and they were investigated using χ^2^ test (Tables 2 and 3). Age of study-victims ranged from 4 to 75 years with a median age of 26 years and mean age of 27.61 years with standard deviation of 10.56 years; the age-frequency curve was found to be asymmetrical. The majority (92.72%) of victims were adults (over the age of 18). In this category, most cases of assaults were of domestic violence (49.63%), followed by sexual assault (17.87%), poisoning (16.87%) and physical assault (12.41%). Minors (below 18 years of age) in the sample studied were the minority (6.28%) and a great majority of them had suffered from sexual assault (81.48%), followed by poisoning (7.42%) and then other assaults (Table 2). Analysis further revealed that the types of violence that occurred to women were significantly associated with their age (χ^2^=5.76, d.f.=1, p<0.05).

**Table 2.** Violence against women (VAW) by age amongst women approaching Sukoon Centre

Violence type	Minors (below 18 years old) n (%)	Adults (18 years and older) n (%)
Domestic violence	0 (0)	200 (49.63)
Sexual violence	22 (81.48)	72 (17.87)
Physical assault	1 (3.70)	50 (12.41)
Poisoning	2 (7.42)	68 (16.87)
Suicide	0 (0)	3 (0.74)
Burn	1 (3.70)	6 (1.49)
Miscellaneous	1 (3.70)	4 (0.99)
Total	27 (100.0)	403 (100.0)

**Table 3.** Violence against women (VAW) by marital status among women approaching Sukoon Centre

Violence type	Married n (%)	Unmarried n (%)
Domestic violence	178 (58.94)	22 (17.19)
Sexual violence	32 (10.60)	62 (48.44)
Physical assault	40 (13.24)	11 (8.59)
Poisoning	45 (14.91)	25 (19.53)
Suicide	2 (0.66)	1 (0.78)
Burn	3 (0.99)	4 (3.13)
Miscellaneous	2 (0.66)	3 (2.34)
Total	302 (100.0)	128 (100.0)

Out of 430 victims, 70.23% were married and the remainder (29.77%) were unmarried (Table 3). Among the married victims, domestic violence was the most common type of assault (58.94%), followed by physical assault (13.24%) and then poisoning (10.60%). Among the unmarried victims, sexual assault was the main type of assault (48.44%), followed by poisoning (19.53%) and then domestic violence (17.19%). The women's marital status was found to be significantly associated with their types of assaults (χ^2^=98.23, d.f.=4, p<0.001).

Table 4 shows that out of 430 violence cases, a great majority of the cases (69.30%) were resolved either by Sukoon directly (35.58%) or with police intervention (33.72%). In some cases, Sukoon also helped victims by providing legal aid. There were 8.61% of victims who received justice through legal courts under the guidance of Sukoon. Despite all this, a considerable number of violence cases were still pending in the courts of law (22.09%) at the time of the end of the study. Of the 430 victims, 334 (77.67%) were identified by the doctors from hospitals and referred to Sukoon after examining them. Fifty-six cases (13.02%) approached Sukoon through the available helpline numbers, and 40 cases (9.30%) were referred to Sukoon as medico-legal cases (Table 4). When the status of the association between sources of victims and intervening agencies for resolving violence cases was tested, it was found to be statistically significant (χ^2^=58.74, d.f.=6, p<0.001).

**Table 4.** Intervening agencies in resolving cases of violence against women (VAW) vis-à-vis their sources of information

Sources of Victims				
Intervening agencies	Sukoon got as a medico-legal case n (%)	By Sukoon directly n (%)	Through screening & counseling by Sukoon n (%)	Total n (%)
Resolved through	1 (2.50)	17 (30.36)	135 (40.42)	153 (35.58)
Sukoon				
Resolved through court of law	9 (22.50)	8 (14.28)	20 (5.99)	37 (8.60)
Resolved through police	8 (20.00)	14 (25.00)	123 (36.83)	145 (33.72)
Cases still pending	22 (55.00)	17 (30.36)	56 (16.76)	95 (22.09)
Total	40 (100.0)	56 (100.0)	334 (100.0)	430 (100.0)

## Discussion

Continuous efforts are made by the Central and the State Governments to empower women, but violence still remains one of the most pressing problems in India. Victims are often uneducated women from low socio-economic status, which increases their possibility of visiting a public hospital. Dealing with cases of violence is a part of healthcare services, and screening of victims should be a routine practice. In already overcrowded Indian hospitals this may often prove challenging. Screening procedures in a hospital increase the possibility of identifying victims of domestic violence,^22^ and it is already accepted by women in Indian healthcare settings^23^. In cases of injury, victims often report to hospitals for medical aid, however, violence resulting in minor injuries often go unreported. A health care system can be a safe and secure environment for women suffering from violence, where they can disclose their experience with confidentiality. The results of the present paper further support the idea of institutional screening and providing support to victims, thus proving that health care systems (hospitals) play a crucial role in response to VAW^24^. Doctors often play a leading role in early identification of VAW, supportive responses, clinical care, and referrals as per the need of the victim^25^. The victims of intimate partner violence trust healthcare specialists in disclosure of abuse^26^. However, the lack of knowledge, practices and support services on the issue makes this challenging^27^. Proper training of health care providers can create a change in their attitude and practices in addressing cases of assault^28^. The large number of cases identified through Sukoon further support the presence of OSCC in hospitals for screening and counseling of victims. The Dilaasa Model also proved that the presence of an OSCC along with active screening has helped in early detection of domestic violence^19^. In this study, age and marital status were found to be the possible risk factors of the types of violence seen, which is similar to the results reported by Babu and Kar^29^. Paul studied the role of socioeconomic factors which were responsible for seeking help by women victims of violence emphasized the role of age, education and religion in seeking formal or informal help against violence^30^. In India, victims while seeking help preferred informal sources like family, neighbors or friends as compared to seeking help from formal sources like police, doctors or lawyers. Contrary to this, most cases (77.7%) came to Sukoon through counseling and screening of the victims from the hospital, which further highlights hesitation of victims in reporting cases of violence. Researchers have proved that victims receiving institutional support reported less violence by their husbands in their follow up visits^31^. This emphasizes the need for counseling and awareness among the victims of violence for proper help and advice to fight against violence. Further, a considerable number of cases were provided help by the police (33.7%). Dealing with violence is a teamwork which requires proper coordination at various levels, and every case needs a different approach to help the victim to fight against violence. The delay in the settlement of cases shows that justice to the victims at the level of judiciary in India is still quite slow. The high number of court cases still pending demonstrate a delay in the judicial process due to various reasons. The Sukoon Crisis Centre also faces some challenges in meeting the needs of victims. There is a need for separate counseling rooms at the Centre to attend different victims at the same time and maintain their privacy. Counselors face a major problem if repeated calls have to be made to the police for any type of intervention. Sometimes, doctors do not refer victims to the OSCC due to a huge rush of patients. Many times, victims of violence do not want to go back to their homes. In such situations, provisions should be made for temporary stay-arrangements at shelter homes. Some initial financial aid should be provided to the victims until the case is taken up or transferred to some other authority.

This study has helped in highlighting the relevance of OSCCs in assisting the victims of violence along with the problems associated with the functioning of these centers. But the study has certain limitations also, as the results of this study are based on the data from only one hospital of Haryana and do not represent the success of all OSCCs in general. However, the success of other OSCCs will differ according to the availability of services and experts in each hospital. Also, the time taken to solve each case is not included in the study, and thus, it is not possible to comment upon this aspect. Further studies with wider samples and demographics are required along with the feedback from the victims so as to ascertain problems they face at various levels, which would help in effective functioning of the OSCCs.

Sukoon provides support to the victim at various levels, which includes providing emergency treatment, followed by treatment to mitigate potentially long-lasting effects of the violence in later stages of life and educating the victims on violence and how to advocate for themselves against any type of violence.

To address the problem of domestic violence more efficiently, concrete changes are required in education and clinical systems. Measures to deal with violence cases ought to be formally included in the study curriculum of medicine and to make screening and reporting cases of violence a part of their responsibility. Domestic violence cannot be curbed by a single specialty of experts—it is a teamwork requiring the skills of different fields, including government policies, hospitals, non-government organizations, police, lawyers and judges. They should be trained in their respective domains to address cases of violence.

To help the doctors in the screening of VAW, a representative of Sukoon (counselor) should be posted in the outpatient door area (OPD) to facilitate referral of victims to Sukoon. Along with doctors, nurses can also play a significant role in dealing with domestic violence cases within hospitals, as they are the people who are in direct contact with patients for the longest period of time, especially in the hospital area.

Helping a victim of violence at Sukoon needs coordination between various agencies like police, doctors and lawyers. This often leads to unnecessary delay of the process at various steps. Thus, a time limit should be decided for helping all cases of violence. Any delay at a certain point should be documented with valid reasons. A strong partnership between a non-government organizations and healthcare workers is required for OSCCs^29^. Victims should be motivated during follow up to make other females aware of such centers and bring any female there if she is experiencing any kind of violence.

Results of this study justify that hospitals can be an ideal place to identify victims of violence and as such, there is a need to establish more OSCCs at hospitals of various levels with their regular monitoring and evaluation. Screening and attending to the victims within the hospital can be a positive approach in identifying and helping them. A regular teaching and training program, along with spreading awareness regarding violence, should be an important activity of every OSCC for women.
